# Seasonal changes in coat colour and sexual size dimorphism in a subtropical ungulate

**DOI:** 10.1098/rsos.250379

**Published:** 2025-09-17

**Authors:** Tania A. Perroux, Alan G. McElligott, George M. W. Hodgson, Kate J. Flay

**Affiliations:** ^1^ Department of Veterinary Clinical Sciences, Jockey Club College of Veterinary Medicine and Life Sciences, City University of Hong Kong, Hong Kong; ^2^ Department of Infectious Diseases and Public Health, Jockey Club College of Veterinary Medicine and Life Sciences, City University of Hong Kong, Hong Kong; ^3^ Centre for Animal Health and Welfare, Jockey Club College of Veterinary Medicine and Life Sciences, City University of Hong Kong, Hong Kong

**Keywords:** body size dimorphism, *Bos taurus*, climate, coat colour, free-ranging, Gloger’s rule, horn length, thermal melanism

## Abstract

Phenotypes reflect the adaptations of organisms to their environments, with common rules defining how coloration and body size should vary based on climate and latitude. Hong Kong (HK) cattle present an opportunity to study these adaptations in one of the very few cattle populations not directly controlled by humans. These cattle are free-ranging in a subtropical climate, characterized by high humidity and temperatures during the wet season, and scarce precipitation during the dry season. We studied seasonal coat colour changes in HK feral cattle, and sexual dimorphism in body size and horn length. We provide the first evidence of seasonal changes in coat colour in cattle, with paler coats being more common in the wet season, while darker coats prevailed in the dry season. These seasonal changes were influenced by temperature, wind speed, solar radiation and body condition. We found that males were larger and had longer horns than females. Our results show a male-biased sex dimorphism in the HK feral cattle. Additionally, our findings suggest that thermoregulation costs drive coloration in these cattle. The phenotypic plasticity we demonstrate in these subtropical feral cattle improves our knowledge of the adaptations of ungulates to their habitat.

## 1. Introduction

Animal phenotypes are visible characteristics of individuals that result from the interplay of genotype and environment. In wild populations, in which genetic and ecological information may be scarce, phenotypes provide an overview of the adaptations of organisms to their environment. Phenotypic traits may differ from previous populations as a result of human-generated selection pressures [1,2] and changes in climatic conditions [3–5]. Description of phenotypes at individual and population level are the foundation of many theories of patterns in ecology such as Bergman's rule (body size associated with temperature and latitude [6,7]) and Gloger's rule (variation in coloration in relation to temperature and humidity [8,9]).

Coats serve multiple functions, including providing thermal insulation by creating a barrier between the skin and the ambient environment, as well as reflecting solar radiation to reduce heat gain [10–12]. Generally, paler coats reflect more solar radiation compared with darker ones, which is advantageous for animals in tropical environments [11]. Conversely, darker coats provide protection against ultraviolet (UV) radiation [13]. As postulated by Gloger’s rule (reviewed by [9]), species inhabiting hot and wet environments, such as tropical and subtropical habitats, are expected to have darker coats [14,15]. This trend has been confirmed in various mammals, notably primates [16,17], wild pigs [13], rodents [18] and more generally in artiodactyls [19] by comparing populations from temperate habitats with populations in tropical and subtropical habitats. However, further complexity emerges when accounting for wind speed impacts on coat insulation [20–24], as well as coat structure [20].

Phenotypic traits can exhibit plasticity, as demonstrated by the periodic moulting of coats in various endothermic species [10,12]. Moulting is influenced by external factors, with photoperiodism (i.e. daylight duration) triggering the initiation of the process, while climate conditions, such as temperature and snow cover, affect the speed of moulting [12]. Additionally, the timing and duration of moulting are governed by internal factors, including age, sex, body condition and reproductive status, all modulated by the endocrine system [10,12]. Predation pressure can be a primary driver of coat coloration, as camouflage decreases predation risk through background matching [25,26]. In environments with significant seasonal contrasts, such as temperate and arctic habitats, these seasonal changes in coloration facilitate adaptations that improve concealment in response to fluctuating vegetation and soil conditions [12,27]. Typically, endotherm coats are darker in summer and lighter in winter, with variations in colour reflecting the balance of eumelanin (black/brown pigments) and phaeomelanin (yellow/red pigments). Seasonal changes in coat colour are expected when the energetic costs of producing a new coat are lower than the costs associated with suboptimal coloration [10].

Sexual size dimorphism typically results from intrasexual competition and/or intersexual mate choice [28–31] and can be expressed in a variety of measurements of body lengths and secondary sexual traits (e.g. ornaments). Although recent meta-analysis revealed that sexual size dimorphism is not as common as previously reported [32], artiodactyl (order Artiodactyla) males tend to be both larger and longer than females [32–34]. In polygynous ungulates, males are larger than females due to intrasexual competition, as larger body size and longer ornaments in males result in higher social status and fitness [33,35,36]. Intersexual selection (i.e. mate choice) for larger ornament in males is also widely documented in ungulates [31]. Several bovid species are characterized by striking sexual dimorphism, particularly in body size [33] and horn shape [37–39], while others have little to no sexual dimorphism [40]. More extreme sexual dimorphism decreases longevity in male bovids [41]. Horns are secondary sexual characteristics displayed by either one or both sexes in bovids [42]. Most bovids grow horns continuously by the proliferation of germinal layer cells [42]. The horns have a diversity of important biological functions, including defence from predators, fighting weapons, courtship and thermoregulation [42–44].

The relationship between phenotypic traits and overall fitness is referred to as phenotypic quality, which reflects the improved survival or reproductive success of individuals with advantageous traits. Therefore, it is expected that these advantageous traits will co-vary. Specifically, survival has been linked to adaptive coat colours (including camouflage and moulting [3–5]) and female body mass [45], while reproductive success is associated with darker coat colours [46,47], the size of ornaments (such as horns and antlers) in males [31,48,49] and females [50], and body mass in males [49,51,52] and females [53].

**Figure 1.**
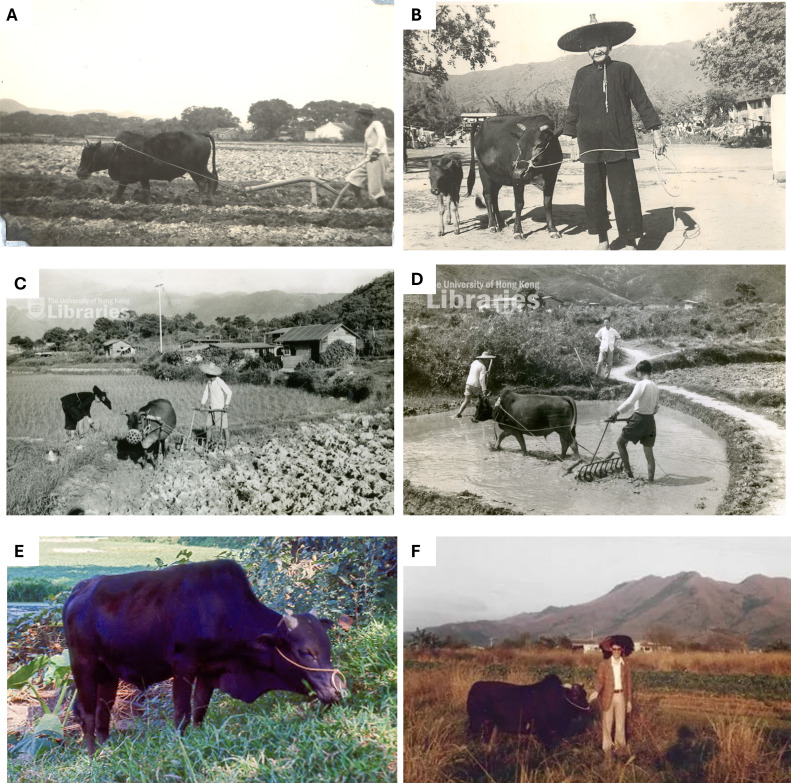
Phenotypes of cattle on farms in Hong Kong before their release. (A) Circa 1930s (photo credit: the Hong Kong Heritage Project [73]). (B) Circa 1950s (photo credit: the Hong Kong Heritage Project [73]). (C) Circa 1962 (photo credit: Hong Kong University [74]). (D) Circa 1963 (photo credit: Hong Kong University [75]). (E) Circa 1966 (photo credit: Malcolm Peaker). (F) Circa 1978 (photo credit: Cliff Crossfield).

There are 1.5 billion cattle globally, with the vast majority managed on farms [54]. It is almost impossible to study cattle phenotypes in populations that are not directly controlled by humans, as there are only a few feral populations. Although it is known that darker cattle display higher signs of heat stress and at lower thresholds than paler coated cattle in agricultural settings [55,56], evidence in feral populations is lacking, and seasonal coat colour changes have not been previously documented in cattle. The feral cattle of Hong Kong (HK) are a genetically distinct mix of *Bos taurus taurus* and *Bos taurus indicus*, with some evidence that they also carry genes from wild Asian bovids [57], similar to other Southeast Asian cattle [58–61]. Originally used as draught animals, these cattle are now regarded as part of the local heritage [62,63]. HK feral cattle are likely to have adapted to match the local environment and were subjected to low selection pressure for production traits even when they were used on farms [57]. While the number of farms raising cattle before their release is unknown, government records [64–72] note that 11 196 to 13 800 cattle were used for draught between 1963 and 1973 and there are records of widows being given cattle after the Second World War [70] (figure 1B). These records also indicate there were 19 herds of dairy cattle from various origins at that time [64–72,76,77]. Combined, this suggests that cattle were widely dispersed across the territory. After their release, some translocations occurred between different herds, including those on islands and on the mainland [62]. Phenotypes of HK cattle on farms were not historically recorded, but photographic evidence suggests a remarkable phenotypic diversity (figure 1), similar to noted recently in the feral population [57]. Currently, there are about 900 feral cattle free-ranging in HK [78].

A number of phenotype characterization methods have been developed for livestock and companion species (e.g. [79–81]), where individuals can be easily restrained for measurements. However, in populations where restraint is more challenging (e.g. large ungulates, wild populations), phenotyping data is often assessed using corpses [82,83] or using invasive procedures (e.g. capture [84]). Non-invasive methodologies, such as measures from photographs (i.e. photogrammetry), are becoming more popular for phenotypic data collection in free-ranging animals (e.g. [46,85–87]), providing valuable information about understudied populations.

Despite the striking phenotypic diversity of these animals, little is systematically known about the phenotypes of feral cattle in HK. We aimed to assess their phenotypes in a non-invasive way, with a focus on coat colour, body size and horn length in respect to seasonality and sexual dimorphism. We predicted distinct seasonal changes in coat colour between the dry and wet seasons, influenced by climate, notably solar radiation and day length, as observed in other species. In the absence of selection pressure for camouflage resulting from the lack of predators, we hypothesized that observed seasonal coat colour changes would be driven by thermoregulation needs and that changes in body condition may reflect the costs of moulting. Additionally, we expected the HK feral cattle to follow the general assumption of sexual dimorphism in artiodactyls, with males being larger and having longer horns than females. We expected male phenotypes to co-vary with traits associated with higher quality (larger body size, longer horns and darker coloration) co-occurring, while female phenotypic traits were expected to vary without presenting patterns, due to higher sexual selection on body size and coloration phenotypes in male than in female large ungulates.

## 2. Material and methods

### 2.1. Seasonal coat colour

#### 2.1.1. Study sites and animals

The HK feral cattle population is currently estimated at 900 individuals [78] found in a variety of environments, from urbanized areas to rural country parks, with some herds on geographically isolated islands. Their main predators, the South China tiger (*Panthera tigris tigris*) and leopard (*Panthera pardus*), have not been observed in HK since the 1940s, and although cases of predation on calves by Burmese python (*Python bivittatus*) and feral dogs have been reported [88], those are uncommon. The Agriculture, Fisheries and Conservation Department (AFCD) of the Government of the Hong Kong Special Administrative Region (HKSAR) provides veterinary care to injured individuals, but routine care is not provided [89]. The AFCD aims to achieve a stable cattle population primarily relying on reproduction control [78,90,91].

We selected 12 herds for longitudinal scoring over a period of 13 months, with a total of 253 cattle scored. These herds were selected based on their relative ease of access, ensuring reliable follow-up, while also maintaining good representation of the population (i.e. herd size, sex ratio, environment type, provisioning, proximity and frequency of interactions with humans). Following initial scoring (i.e. August 2022 during body size measurements), we visited each of the 12 selected herds once a month from January 2023 to January 2024 to obtain individual coat colour scores over time. A total of 2371 coat colour scores were recorded, with each individual scored 1 to 14 times (mean = 9.37 ± SD 4.43). Individuals were identified based on a photo-catalogue (recording traits such as horn shape and scarring to the body and ears) and, when available, visual identification ear tags applied previously by the AFCD. This photo-catalogue is extensively used in various research conducted in this population [92–94].

#### 2.1.2. Measures

**Figure 2.**
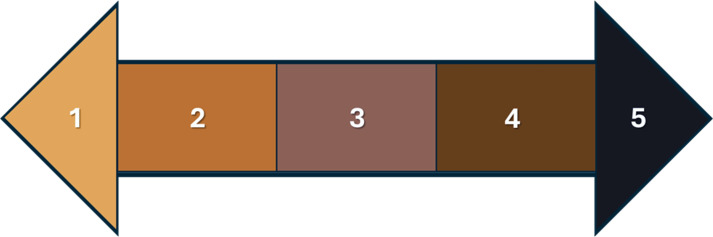
Coat colour scale used to characterize coat colours in Hong Kong feral cattle with 1 corresponding to palest coats, 2 for coats scored as red, 3 for grey, 4 for dark and 5 black (the darkest coat colour). This colour scale is adapted from Stoner [19].

We scored coat colour while maintaining a minimum distance of 10 m from the cattle. Coat colour was scored as a qualitative and subjective measure, controlling for lighting conditions and observer subjectivity influences by using distinct categories of score [17,46], using a colour chart adapted from Stoner [19]. To ensure data reliability, scores made in August 2022 were checked by three independent observers, and the scores were discussed until all observers reached an agreement. Initially, a seven-colour scale was used (electronic supplementary material, figure S1), with attempts to distinguish eumelanin and phaeomelanin pigments, but discrepancies between the four observers in August 2022 led to refinement of the scale into the five-colour scale described in figure 2. Our final colour chart consisted of five coat colours: pale, red, grey, dark and black (figure 2) as described by Stoner for artiodactyls [19] and was used for all scores assessed in this study. This colour chart was comprehensive and covered all coat colour encountered in the field. We used a reference catalogue of cattle with known coat colours under different lighting conditions (vegetation, cloud cover and sunshine variations) photographed between July and August 2022 to guide scorings (electronic supplementary material, figure S2). All coat colours scored from January 2023 to January 2024 were estimated live in the field by a single observer (TP) to reduce subjectivity of scoring. We used this colour chart to assess three body parts: fore, middle and hind [40,47]. Then, we used the mode of those three body part coat colours to obtain each individual’s coat colour per month. Individuals for which coat colour in all three body parts differed were not retained for that month; this was the case for eight individual cattle scores. Further description of the eight data points removed is presented in electronic supplementary material, table S1. Moulting is known to follow patterns across the body which are well described in several species [12], but such prior knowledge was not available for our study animals. Hence, these discrepancies of colour across body part may simply reflect the moulting process but could not be analysed in the present study. To assess coat colour changes, coat colours were replaced by an ordinal scale, with palest coats scored 1 and darkest scored 5 (figure 2).

Each individual received a body condition score (BCS) at the time of coat colour scoring based on a modified visual 9-point scale, with 1 being the thinnest individuals (low body condition) and 9 the fattest (high body condition). This scale was adapted to the HK feral cattle based on beef cattle BCS [95,96]. To control for the subjective nature of body condition scoring, training was conducted to ensure good scorer reliability (for 6 weeks from 1 April 2022 to 5 May 2022), with body condition scored once a week by a single observer on cattle in a single herd (*n* = 60 individuals). BCS scores were expected to be highly correlated from 1 week to the next, and therefore the scorer trained until BCS from 1 week to the subsequent reached a significant correlation of more than *ρ* = 0.8 (based on Spearman’s rank correlation) for three consecutive weeks. This same observer scored all BCS used in this study.

#### 2.1.3. Climate data

We extracted monthly averages for mean daily temperature (in °C), mean daily water vapour pressure (in hPa), total rainfall (in mm), total bright sunshine duration (in hours), mean daily global solar radiation (in megajoule per square metre), mean daily wind speed (in km h^−1^) and mean daily ultraviolet (UV) index from the Hong Kong Observatory database [97]. Seasons were defined based on a principal component analysis of climate data from HKO from January 2015 to July 2022, hence independent from our data collection period. Using a hierarchical cluster analysis, we confirmed that HK subtropical weather followed four periods: the dry season (from December to February); the wet season (from May to October); and two intermediate seasons (one from dry to wet season, March to April; one from wet to dry season, November). Climate during our sampling period is summarized in electronic supplementary material, table S2.

#### 2.1.4. Statistical analysis

We performed descriptive statistics using JASP software [98], while further tests were performed using R [99]. All scripts and datasets are provided on OSF database [100].

As our data deviated from normality (as tested by Shapiro–Wilk test), we analysed the repeatability of scores between the years with a paired (per individual) Mann–Whitney signed-rank test, comparing scores made in August 2022 with scores in [101] scores made in January 2023 with scores in January 2024. Seasonality of coat colour incidence was investigated using an adapted cosinor regression model with coat colour as an ordinal response variable and months as the predictor variable, modelling a sinusoidal function in a general linear model (similar to [102]) adapted to our ordinal response variable using ordinal logistic regression (OLR) with the function ‘clm’ (package ‘ordinal’). Differences in distribution of coat colours between the sexes were analysed using a *χ*² test and adding months as a layer in the contingency table. Coat colour was transformed into a continuous scale from −4 to 4 to form the variable ‘magnitude of coat colour change’; this scale reflects an individual’s changes in coat colour from one month to the subsequent throughout our 13 months of data collection across 12 herds, with negative values for coats getting paler and positive values for coats getting darker, while 0 indicates no changes in coat colour.

The distribution of coat colours in the population was analysed using OLRs. A first OLR compared the distribution of coat colours with the magnitude of coat colour change. Then, a second OLR was used to test the impact of BCS and climate variables (mean daily temperature, mean daily water vapour pressure, total rainfall, total bright sunshine duration, mean daily global solar radiation, mean daily wind speed and mean daily UV index) from the month of the score and the month before the score on the distribution of coat colours. A backwards elimination procedure was conducted, with variables with the highest *p*-values removed stepwise until only significant variables remained. Multicollinearity of the predictors was checked using the variation inflation factor (VIF) with the function ‘vif’ (package ‘car’), with values below 5 considered as conforming to OLR assumptions. A Brant test was conducted to ensure that the proportional odds assumption was respected, using the ‘brant’ function (package ‘brant’), with *p*-values above 0.05 indicating that the assumption holds. Predicted probabilities (PP) were extracted from the OLR model to facilitate interpretation of the results, using meaningful points on significant predictors (i.e. using five integers along the observed predictor natural range).

Coat colour change was analysed using an OLR, removing extreme coat colour changes (−3 and 3) as those scores were uncommon and did not conform to the proportional odds assumption. Coat colour change was used as the dependent variable (outcome). Sex and climate variables (mean daily temperature, mean daily water vapour pressure, total rainfall, total bright sunshine duration, mean daily global solar radiation, mean daily wind speed and mean daily UV index) from the month of the measurements and the previous month were entered as independent variables. Variables with high correlations were removed to ensure the assumption of absence of multicollinearity was met, this was the case for five variables (water vapour pressure from the month before scoring, and UVs, water vapour pressure, rainfall and temperature of the month of scoring). Then, a backwards elimination procedure was conducted, as described in the previous paragraph.

Results are presented using odd ratios (OR) and corresponding confidence intervals (CIs), which can be interpreted as follows. For continuous predictors, ORs greater than one indicate that the higher categories (darker coats) are more likely as the predictor increases, while ORs that are equal or less than one indicate that the lower categories (paler coats) are more likely as the predictor increases. For categorical predictors, ORs greater than one indicate that the higher categories are more likely at the alternative level than at the reference level of the predictor. ORs and corresponding CIs above four indicate strong association between the predictor and the response variable, while ORs and CIs between 1.5 and 4 probably indicate an effect mitigated by other predictors in the model. ORs below 1.5 indicate low to no associations.

### 2.2. Sexual size dimorphism

#### 2.2.1. Study sites and animals

**Figure 3.**
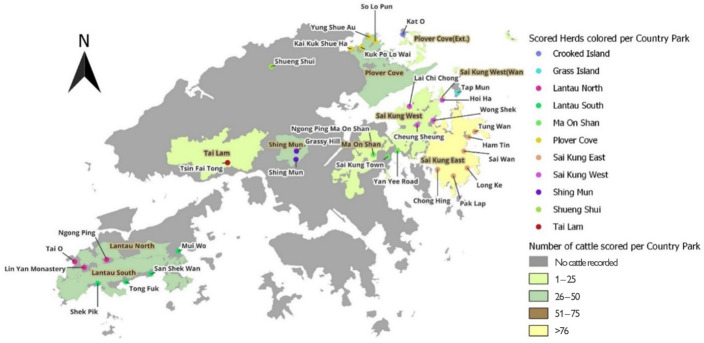
Map of the herds sampled for body size measurements in Hong Kong SAR. The colours represent the number of cattle scored in country parks. Country parks were defined following the Agriculture and Fisheries Conservation Department (AFCD) of Hong Kong SAR. Country Park names have brown labels, while white labels indicate herd names. Dot colour indicates country park membership.

We studied 30 different cattle herds (figure 3), scoring one to 43 individuals in each herd (electronic supplementary material, table S3), with a total of 317 individuals scored (about a third of the population). We visited each location at least once and up to three times. Herds were distributed over 11 different country parks (*n* = 5–117 individuals per country park), with country parks defined by the AFCD. We collected data during HK’s wet season, between 27 July 2022 and 30 October 2022.

We excluded calves and only scored adult cattle as recommended for description of breed populations [103]. Our exclusion criteria defined calves following two criteria: (i) absence of apparent horns or horns detached to the skull (horn tips moving separately to jaw movements) and (ii) similar appearance to same-aged individuals (electronic supplementary material, figure S3). We scored all cattle meeting the inclusion criteria (i.e. adult cattle) and gave each a unique identifier; either its tag as provided by the AFCD, or the name of the herd followed by the number of individuals found that day (e.g*.* the fourth individual found in Tai O was identified as TO4), unless the animal had been named for other studies.

#### 2.2.2. Measures

**Figure 4.**
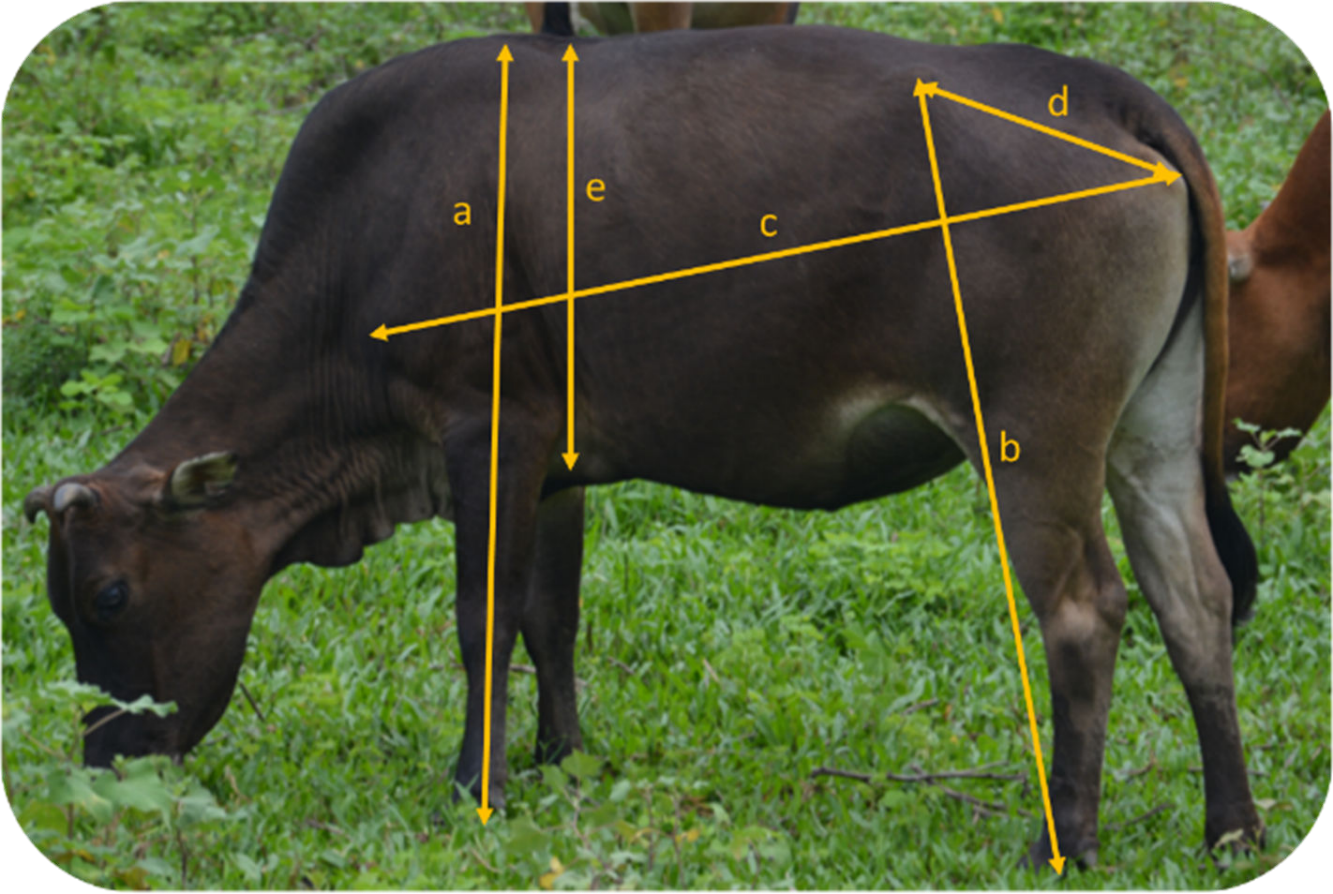
Different measures used to assess body size based on pictures of Hong Kong feral cattle: (a) wither height, (b) hip height, (c) body length, (d) hip width and (e) chest depth.

Side view photos were taken of each individual to assess body size measures [104–106]. These photos could be taken without restraint, and digital analysis of photographs also decreases intra- and inter-observer variability compared with measures made with a tape, particularly for linear measures [107]. We used five measures: wither height, hip height, body length, hip width and chest depth [108,109] (table 1; figure 4). To ensure reliability of the measures, we used a triangulation method: the scorer took a photo of the cow and a field assistant. The distance to the animal was decreased slowly until equal distance between cow, scorer and field assistant was met, at which point the scorer took the photo. As soon as the photo was obtained, the scorer and field assistant stepped away from the cow. As this was not possible for all individuals (i.e. not able to safely approach and/or the animal repeatedly moved away), body size measures were not recorded for all cattle, with measurements obtained for 122 females and 100 males. Using the photos, we took measures with Prime Ruler Android application (Grymala, Warsaw, Poland). We used the real height of the assistant divided by the height given by the app as a factor to correct the application measures as follows:


$$RR_{cattle}={\text{PR}}_{cattle}*\frac{{\text{PR}}_{assistant}}{{\text{RR}}_{assisant}}$$

**Table 1.** Body size measures used to describe the Hong Kong feral cattle.

body size measure	anatomical definition
wither height	from wither to hoof of fore limb
hip height	from tuber coxae to hoof of hind limb
body length	from shoulder joint to tuber ischii
hip width	from tuber coxae to tuber ischii
chest depth	from withers to brachial pit of fore limb

where RR = real measure estimation and PR = Prime Ruler measurements.

We scored horn length by visual comparison with ear length along three categories: shorter, similar or longer than ear length. We developed this method to enable scoring horn phenotypes without (i) restraining individual cattle to obtain tape measurements, and (ii) using portrait photographs that would require the scorer to stand in front of the cattle at a potentially unsafe distance. Though photogrammetry has been used for ornament measurements in other ungulates [48,85], limited visibility in some of the habitats of HK cattle (e.g. forest, wetlands, villages), limited our ability to obtain portrait images. For this reason, we used a categorical rather than numerical variable as horn length measurement. Horn length was scored for all study animals using both horns (170 females and 147 males). We accounted for broken and asymmetrical horns by adding them as a category, resulting in a total of five categories.

#### 2.2.3. Statistical analysis

As all measures of body size were highly correlated (based on Spearman’s correlation, with all correlations *ρ* > 0.54 and *p* < 0.001; electronic supplementary material, table S4), we used a PCA (function ‘PCA’, package ‘FactoMineR’ [110]) to obtain a summary numerical variable for body size. The first principal component (PC1) of the PCA was selected, as it explained 77.81% of the variance, had an eigenvalue of 3.89, and was significantly positively correlated to all five measures of body size (*p* < 0.0001). We used a linear mixed-effect model (LMM; function ‘lmer’, package ‘lme4’ [111]) to assess for sexual dimorphism of body size; body size was used as the dependent variable and sex as the independent variable. Location nested within country park was used as a random factor. The significance was obtained using Satterthwaite’s method for single term deletions (‘drop1’ function, package ‘stats’).

Sexual dimorphism index (SDI) is a measure developed by Lovich & Gibbons [112] to quantify body size difference between the sexes. It was adapted for ruminants [33,113,114] as follows:


$$\frac{average\;of\;male}{average\;of\;female}$$

In our analysis, male was the larger sex used in the formula. This measure was calculated to describe sexual dimorphism in all five body measurements. Sexual dimorphism of horn length was assessed using a Pearson’s chi-squared test (function ‘chisq.test’, package 'stats') to compare the distribution of each category of the variable horn length between males and females.

### 2.3. Coat colour, body size and horn length phenotype patterns

#### 2.3.1. Data preparation

Two datasets were prepared: one for females and one for males to account for sexual dimorphism. The mode coat colour of dry (December to February) and wet (May to October) seasons were combined with horn length and body size (PC extracted from PCA of body size measurements), These data were available for 68 females and 54 males. Modes from intermediate seasons were not selected as they are much shorter in duration than dry and wet seasons and due to high number of missing values in intermediate seasons.

#### 2.3.2. Data analysis

The same analysis was conducted for males and females separately. First, the relationship between coat coloration in the wet and dry seasons and horn length were analysed using contingency tables (*χ*^2^). Then, we investigated the differences in coat coloration in the wet and dry season and horn length in relation to body size using type III analysis of variance (ANOVA; function ‘aov’). Specifically, body size PC was used as the dependent variable, while dry season coat colour, wet season coat colour and horn length were used as independent variables. While we acknowledge the ordinal nature of our coat colour variables, they were coded as categorical data to investigate the incidence of each coat colour per season separately. Assumptions were tested using a Shapiro test to ensure normality and a Bartlett test to ensure homoscedasticity. Tukey tests were used to conduct post hoc comparisons, using a Bonferroni correction for *p*-values to adjust for multiple comparisons.

## 3. Results

### 3.1. Seasonal coat colour

**Figure 5.**
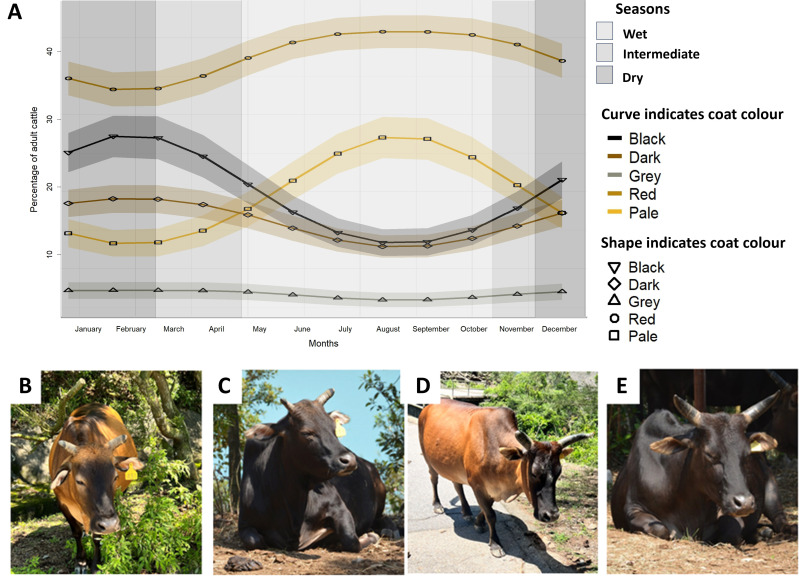
(A) Predicted monthly variation of coat colour of the Hong Kong cattle. (B–D) Examples of coat changes within the same individual: female (tagged 431) in the wet season (B) and in the dry season (C); and male (tagged 17) in the wet season (D) and in the dry season (E). Coat colour was scored based on a colour chart. Predicted percentages and corresponding confidence intervals (shaded) are based on the probabilities extracted from cosinor analysis.

Red coats were the most common coat colour in all seasons, while grey coats were the least common (*χ*² = 594.28, *p* < 0.0001; table 2; figure 5). On average 31% (95%CI = 29.33–33.80) of individuals changed coat colour each month, though extreme changes were uncommon. While our methodology would identify individuals that go from black to pale (or pale to black), this was never observed, and extreme changes (−3 and 3) accounted for less than 1% of individual changes. Specifically, 21% of males and 24% of females did not change coat colour throughout the study period, while only 11% of females and 13% of males changed from one extreme coat colour to another within 1 year (table 3). Coat colours followed seasonal patterns, with no difference between the scores made in August 2022 and 2023 (*W* = 553.50, *p* = 0.40) and in January 2023 and 2024 (*W* = 471.00, *p* = 0.98). Seasonal changes in coat colours were further confirmed by significant cosine (OLR; *β* = −0.13, *p* = 0.01) and sine functions (OLR; *β* = −0.43, *p* < 0.0001), indicating that incidence of each coat colour varied seasonally (electronic supplementary material, table S5), as illustrated in figure 5.

**Table 2.** Distribution of coat colour of the Hong Kong feral cattle in the dry, intermediate and wet seasons.

season	coat colour	counts	frequency (%)	95% CI		
dry (686 scores)	pale	91	13	10.93	—	16.00
	red	267	38	35.34	—	42.62
	grey	28	4	2.83	—	5.83
	dark	133	19	16.60	—	22.51
	black	167	24	21.28	—	27.69
intermediate dry to wet (317 scores)	pale	43	13	10.22	—	17.77
	red	103	32	27.57	—	37.83
	grey	29	9	6.44	—	12.82
	dark	63	19	15.85	—	24.61
	black	79	24	20.47	—	29.96
wet (1184 scores)	pale	295	24	22.53	—	27.45
	red	449	37	35.20	—	40.72
	grey	94	7	6.53	—	9.61
	dark	156	13	11.36	—	15.22
	black	190	16	14.06	—	18.24
intermediate wet to dry (170 scores)	pale	38	22	16.74	—	29.18
	red	68	40	32.93	—	47.50
	grey	6	3	1.62	—	7.48
	dark	27	1518	11.15	—	22.12
	black	31		13.15	—	24.71

**Table 3.** Coat colour changes in female (*n* = 99) and male (*n* = 101) Hong Kong cattle categorized based on the individual coat colours recorded within the 13 months of scoring (January 2023 to January 2024).

	females (*n* = 99)		males (*n* = 101)	
	frequency (%)	95% CI	frequency (%)	95% CI
only one colour scored	24	16.86–33.54	21	14.84–30.78
both black/dark and pale scored	11	6.31–18.80	13	8.43–21.93
*transition from one extreme coat colour to another through grey or red*	9	4.85–16.38	13	8.43–21.93
both pale and grey scored	6	2.80–12.59	2	1.01–8.37
both pale and red scored	26	18.59–35.70	23	16.51–32.92
both black/dark and grey scored	8	4.15–15.14	6	3.39–13.62
both black/dark and red scored	24	16.86–33.54	23	16.51–32.92

Note: Only individuals for whom scores were not interrupted for more than three consecutive months were included. As nine females and seven males transitioned from extremes to both red and grey coat colours, they were included in both categories. Black and dark coats were considered together as they were identified as following similar patterns.

There were significant differences between male and female coat colour distribution (*χ*² = 156.20, *p* < 0.0001). Females were in general paler, with red being the most common coat colour in females (44%, 95%CI = 41.59–46.95), while black coats were the most common in males (30%, 95%CI = 28.07–33.67). These differences were not consistent throughout the year, with significant sexual differences in coat colour distribution between males and females in eight months (*p* < 0.05; electronic supplementary material, table S6), while coat colours in May (*χ*² = 8.63, *p* = 0.07), June (*χ*² = 2.96, *p* = 0.56), September (*χ*² = 6.46, *p* = 0.16) and October (*χ*² = 8.92, *p* = 0.06) did not differ between males and females.

Individuals with darker (black and dark) coat colours were more likely to change coat colour than cattle with red, grey and pale coats (OLR; OR = 1.80, 95%CI = 1.57–2.06, *p* < 0.0001). Coat colour was predicted by the monthly average of the mean daily temperature from the month before the measurement (OLR; OR = 0.93, 95%CI = 0.92–0.94, *p* < 0.0001; electronic supplementary material, table S7) and by the BCS at the time of the measurement (OLR; OR = 0.80, 95%CI = 0.71–0.89, *p* = 0.0001; electronic supplementary material, table S8). Following months of lower temperature (15°C), the incidence of darker coats was higher (PP; dark = 0.19; black = 0.30), while following higher temperatures (35°C), the incidence of paler coats was higher (PP; pale = 0.32; red = 0.40). Similarly, the incidence of paler coats was higher in individuals with higher body condition (BCS 7, PP; pale = 0.28; red = 0.39), while in individuals with lower body condition (BCS 3) had higher incidence of dark coats (PP; dark = 0.17; black = 0.23). The interaction between BCS and the temperature from the previous month could not be computed as it increased VIF/multi-collinearity above 60, making it unsuitable for interpretation.

Coat colour changes were significantly explained by wind speed and mean daily global solar radiation in the month of scoring and temperature of the month before scoring, though odd ratios indicate weak associations (OR close to 1). Specifically, cattle coats were paler in months with lower wind speed (LM; OR = 1.05, 95%CI = 1.01–1.08, *p* = 0.003) and with longer duration of global solar radiation (LM; OR = 0.91, 95%CI = 0.86–0.96, *p* = 0.001). Cattle coats were also paler following months of higher temperatures (LM; OR = 1.04, 95%CI = 1.01–1.07, *p* = 0.006).

### 3.2. Sexual size dimorphism

**Figure 6.**
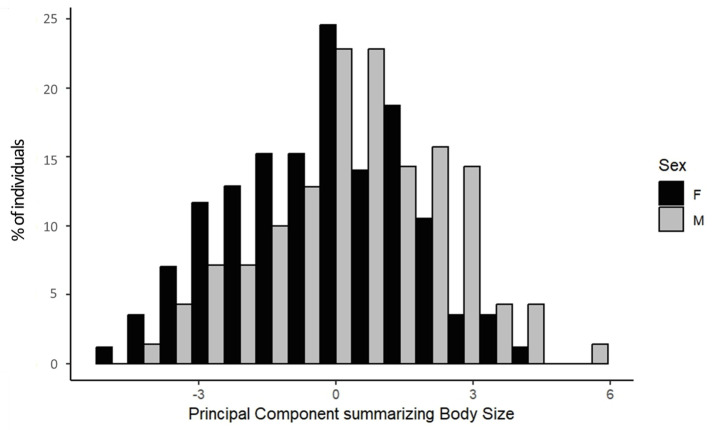
Body size of 100 male (light grey) and 122 female (black) Hong Kong feral cattle. Body size is expressed as a principal component (unitless) obtained from a principal component analysis summarizing five measures of body size: wither height, hip height, body length, hip width and chest depth.

Males were significantly larger than females (LMM, *p* = 0.002; figure 6) in all body size measurements as indicated by the SDI (table 4). While cattle were dimorphic, their dimorphism is moderate, with a mean SDI of 1.07, ranging from 1.05 for hip height and body length to 1.1 for chest depth.

**Table 4.** Body size measurements (expressed in centimetres) and principal component (unitless) obtained from a principal component analysis summarizing the five measures of body size for 100 male and 122 female Hong Kong feral cattle. Sexual dimorphism index (SDI) is given for all five body size measurements as the difference between the sexes ($\frac{average\;of\;largersex}{average\;of\;smallersex}$).

		mean	minimum	maximum	SDI
wither height	F	101.65	66.34	146.01	1.08
	M	110.58	67.96	156.21	
hip height	F	95.20	51.29	144.50	1.05
	M	100.88	30.92	139.08	
body length	F	112.10	70.42	161.19	1.05
	M	118.48	49.19	174.87	
hip width	F	30.55	12.67	61.22	1.07
	M	32.97	10.16	52.39	
chest depth	F	55.54	7.14	91.35	1.1
	M	61.43	14.51	92.40	
PC1	F	−0.33	−5.56	4.75	
	M	0.38	−4.77	6.10	

**Figure 7.**
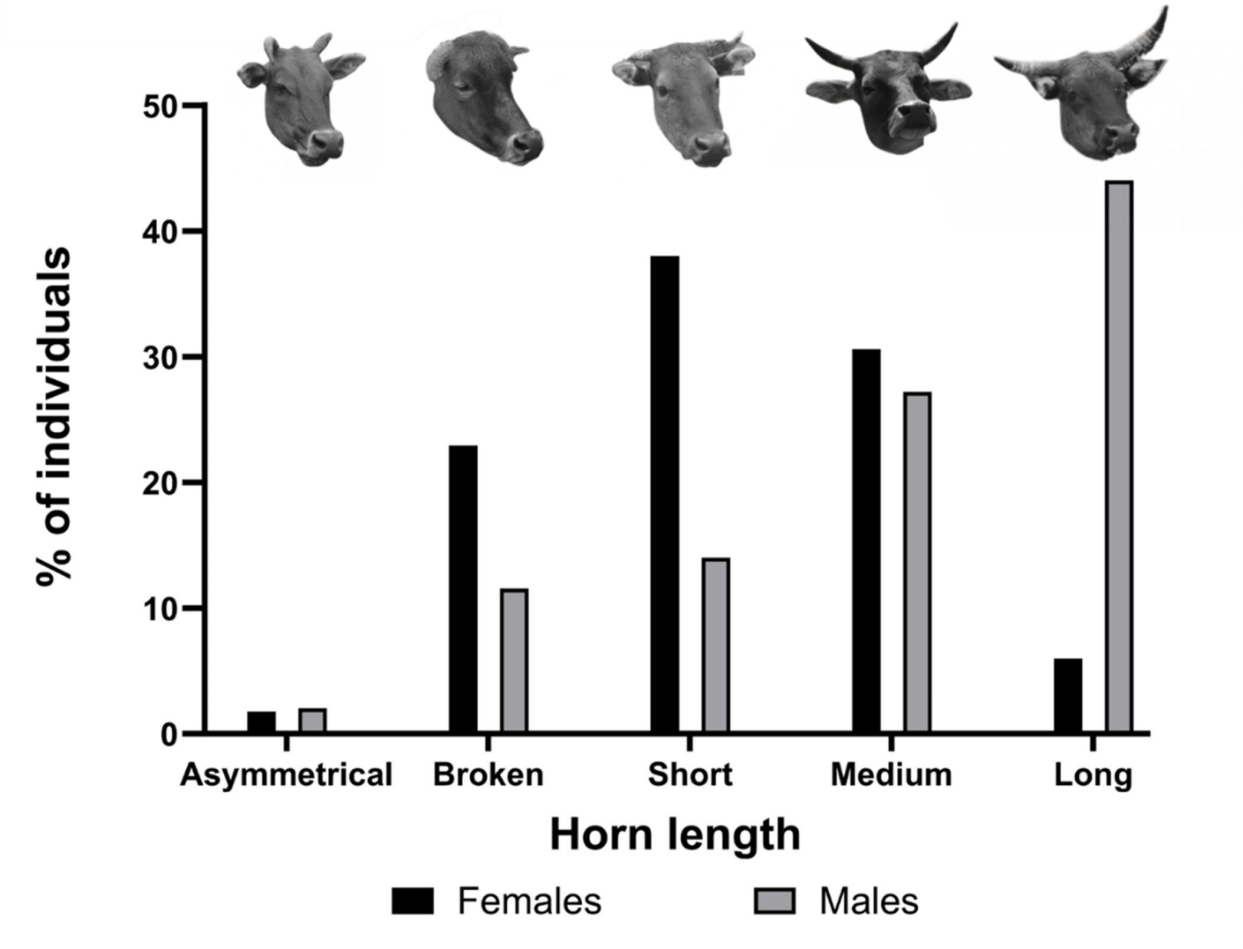
Distribution of horn lengths in 147 males (grey) and 170 females (black) Hong Kong feral cattle. Horn length was measured visually by comparing it with ear length.

Males had longer horns than females (*χ*² = 68.52, *p* < 0.0001; figure 7). While short horns were the most common horn type in females (38%, 95%CI = 21.26–45.72), long horns were the most frequent horn type in males (44%, 95%CI = 36.43–52.29). Asymmetrical horns were uncommon in both males (2%, 95%CI = 0.69–5.82) and females (1%, 95%CI = 0.60–5.05). Females were more likely than males to have broken horns (22%, 95%CI = 17.26–29.81, and 11%, 95%CI = 7.34–17.73, respectively).

### 3.3. Coat colour, body size and horn length phenotype patterns

Neither coat coloration in the dry nor the wet season were associated with horn length in males (*χ*^2^; dry season, *p* = 0.40; wet season, *p* = 0.37) and females (*χ*^2^; dry season, *p* = 0.21; wet season, *p* = 0.61). Female body size was not associated with horn length (ANOVA, *p* = 0.29) nor coat colour in the dry (ANOVA, *p* = 0.13) or wet season (ANOVA, *p* = 0.18). The variance of male body size was associated with horn length (ANOVA, *p* = 0.05), though the pairwise Tukey test did not reveal any association between mean body size and horn length categories (*p* > 0.05 for all pairwise comparisons). However pairwise comparisons may be limited due to some horn shapes (i.e. asymmetrical and broken horns) being much less observed than others. Body size of males was not associated with coat colour in the dry (ANOVA, *p* = 0.11) nor wet season (ANOVA, *p* = 0.36).

## 4. Discussion

We provide the first phenotypic analysis of seasonal coat colour changes and sexual dimorphism in body size and horn length in the HK feral cattle. We present the first evidence of seasonal coat colour variation in cattle, with a third of individuals showing coat colour changes every month. Coat colours were predicted by body condition and the temperature from the previous month, while the magnitude of seasonal coat colour change was predicted by wind speed, temperature and solar radiation. HK feral cattle are sexually dimorphic, with males being darker, larger and having longer horns than females. Additionally, females are more likely to have broken horns than males. As global temperatures rise and weather patterns shift, many species are predicted to face important imbalances in thermoregulation (e.g. [115]), driving phenotypic expression to adapt to increasingly unpredictable environmental conditions. By understanding the pressures prey populations undergo in the absence of predators and the key role of phenotypic plasticity in their ability to adapt to changing environment, we can improve population dynamics projections.

HK cattle exhibit seasonal changes in coat colour, with more individuals displaying paler coats in the wet season and darker coats in dry season. Thermoregulation appears to be the primary driver of seasonal coat colour changes in HK cattle, influenced by wind speed, temperature and solar radiation, as seen in other prey species facing high energetic costs from climate factors [27,116]. Darker coats are generally better suited to tropical and subtropical environments, aligning with Gloger’s rule [9,16,18]. However, the thermal melanism hypothesis predicts that lighter coats can help regulate heat gain and water loss in hot and humid climates [9,117]. For instance, in springbok (*Antidorcas marsupialis*)*,* individuals with paler coats thrive in summer, while those with darker coats are better suited to winter climates [118]. Heat stress is a significant welfare concern in farmed cattle, negatively impacting behaviour and physiology, which can compromise long-term health when climatic conditions exceed cattle thermal neutral range [115,119,120]. Darker cattle breeds tend to suffer more from excessive heat loads compared with breeds with paler coats [56,121,122]. Similarly, coat colour variation within breeds leads to individual differences in heat load, with black coated Holstein cattle having higher surface temperature than white cattle [55,123]. Our findings show that darker individuals have lower body condition, suggesting a higher cost associated with darker coats, in alignment with previous findings in dairy cattle [124]. Genes related to UV protection and heat tolerance have been identified in other Chinese cattle breeds [59,60,125], which may explain the lack of effects of UVs that we observed here, highlighting the ability of HK cattle to tolerate various UV index levels. The association we found between wind speed and coat colour, with paler coats during months of lower wind speed, corroborates previous findings in cattle highlighting higher heat load in cattle with darker coats at low wind speed while no differences were found at higher wind speed [21]. As wind speed appears lower in wet compared with dry season (electronic supplementary material, table S2), this result indicates that wind speed may be a relevant weather variable driving seasonal changes in coat colour and lowering cost of darker coats at higher wind speed levels (i.e. dry season). Further experimental tests are needed to investigate the costs of different coat colours across seasons and weather variables. Our findings suggest a complex interaction between coat reflectance, penetrance and insulation [20,21]. Overall, our results support the thermal melanism hypothesis, as we found that temperature, wind speed, and solar radiation influence seasonal coat colour changes.

Our findings provide the first evidence of seasonal changes in coat colour in a subtropical cattle population. Coat colour changes in cattle have been previously associated with age [126] and with nutritional deficiencies, with for instance copper deficiency being associated with paler coats in farmed cattle [127]. The association we found between darker coats and lower body condition in HK cattle highlights that it is unlikely to be related to nutritional deficiency and is probably associated with seasonal patterns. Additionally, repeatability of scores across the years supports the idea of seasonal patterns of coat colour changes in HK cattle. In tropical ungulates, paler coats could be expected in the dry season for camouflage against vegetation, while darker coats in the wet season provide background matching for darker soil and foliage (see for instance [13]). However, our findings do not support these expectations. Coat colour variability is influenced by a trade-off between thermoregulation and predation risk [10,12,116]. As HK feral cattle were farmed (and thus probably protected by farmers) when predators were still present in HK, there may have been little to no pressure from predation risk on this population historically. Besides, artificial selection in domestic population has been suggested to select for anti-camouflage colorations [128,129]. Hence, coat coloration of HK cattle may not serve a function as an anti-predatory response, resulting in it not matching the background colours as would be expected in other free-ranging ruminants with high predation pressure. Absence of impact of UV on coat colour changes suggest a moulting pattern rather than a bleaching effect driving seasonal changes [12], but quantification of melanin levels in pelts are required for confirmation.

We found that darker coats were present in all seasons, although these were more common in males and in the dry season. If darker coats impose higher thermoregulatory costs, their persistence in subtropical environments like HK raises questions. Darker coats correlate with higher androgens levels and reproductive success [46,47,56,130] as well as enhanced heat dissipation through skin evaporation in Holstein cows [55,131]. Variations in pigmentation relate to behavioural differences [11,132], with darker mammals often exhibiting greater dominance, fertility, aggression and sexual activity [133–136]. In giraffes (*Giraffa camelopardalis*), darker males were more likely to live in smaller social groups and to spend more time alone, reflecting different sexual strategies between paler and darker males, with coat colour playing a role as a signal of competitive ability [137]. Similarly, in eland (*Tragelaphus oryx*), acquiring a higher social status led to darkening of facial coloration, highlighting a social function of phenotypic plasticity in coat colour in ungulates [138]. However, contrasting evidence exists with paler male Himalayan tahr (*Hemitragus jemlahicus*) being larger, higher ranked and having a higher reproductive success than their darker conspecifics [139–141]. Though cattle can discriminate long wavelengths from medium wavelengths [142] suggesting they would be able to discriminate red-based coat colours, further evidence on their ability to discriminate conspecifics based on coat colour and to adjust their behaviour is lacking, but may explain why darker coats persist in this population.

While the coat colour of HK cattle is plastic and exhibits seasonal patterns, most individuals show limited variation in coloration. Cattle with extreme coat colours are more likely to moult, which occurs when the cost of producing a different coat is lower than maintaining a suboptimal coloration [10]. Consequently, cattle with extreme coats may face higher costs when their pelage becomes mismatched, increasing the likelihood of moulting. Similarly, in springbok, extreme coat colours (black and white) were rare, probably due to variations in thermoregulation costs [118]. Extreme colours can yield both significant benefits and higher costs, leading most individuals to exhibit average traits that balance these factors. This is further supported by the low percentage of individuals changing from an extreme to another (11% in females and 13% in males), while most individuals changed from extreme to intermediate values (table 3). While paler coats can reduce heat stress, darker individuals often enjoy reproductive and immune advantages [9,130,134]. Our study addresses a simplified version of Gloger’s rule, examining pigmentation as a whole; however, melanin pigments (i.e. eumelanin and phaeomelanin) are known to respond differently to climatic conditions [9,117]. Further investigation into melanism in feral cattle could enhance our understanding of how climate and thermoregulation costs influence the prevalence of coat colours.

In artiodactyls, males tend to be both larger and longer than females [32,33], similar to our findings that HK feral bulls were on average larger in all body size measures than HK feral cows. Domestication in bovids has led to greater sexual dimorphism with larger males [34,113]. Among cattle, milk breeds are the most sexually dimorphic, while draught breeds are the least dimorphic [114]. Additionally, bovid species from the tropics and subtropics are less sexually dimorphic than bovids from temperate zones [114], with sexual dimorphism index (SDI) in South Asian bovids (including zebus *Bos taurus indicus*, gayal *Bos frontalis* and Asian buffaloes *Bubalus bubalis*) averaging 1.2 while it averages 1.53 in taurine cattle breeds associated with temperate climates. Hence, the moderate sexual dimorphism of HK feral cattle (1.07) may result from their South Asian origin and their genetic similarity with other South Asian bovids, along with their history as draught animals. Thus, HK feral cattle not only share genetic relatedness to Asian bovids [57], we now show they also share phenotypic similarities through moderate sexual dimorphism.

Male bovids usually have longer horns than their female counterparts [34,43,44], as we found in the HK cattle. Sexual selection has been shown to condition horns in males and females differently [44,143]. Horns are primarily used as weapons in male–male competition [43,144–146], resulting in secondary involvement in mate selection, with females favouring males with longer horns in several bovid species [35,144]. Hence, males may benefit from investing resources into growing long horns to improve their reproductive success, while females may favour other functions such as defence against predators, as found in other bovid species [143]. Female HK feral cattle are more likely to have broken horns than males, which may result from differences in behaviour or susceptibility to environmental factors between males and females but is currently unknown.

Contrary to our expectations, we did not find any clear pattern of co-variation between phenotype traits in either male or female HK cattle, similar to many domestic cattle breeds (as reviewed in [56]). While we expected traits associated with phenotypic quality in males (darker coloration, larger body size and longer horns) to co-vary, our results did not show this. However, linear measurements of body size in cattle were previously found to be unrelated to coat colour [56,147]. Additionally, while we provide a proxy of body size through several linear measurements (wither height, hip height, body length, hip width and chest depth), phenotypic quality has often been associated with body mass (see for instance [45,51,53]) rather than body height. The lack of association we found between traits related to phenotypic quality in HK cattle may also arise from the weak relationships between fitness and secondary traits in ungulates with low sexual dimorphism, which probably applies to HK cattle [148]. Further, while evidence in several deer species suggest a positive association between antler size and fitness (see for instance [31,49,149]), similar evidence in ungulates with permanent ornaments, like cattle horns, show contrasting results. In wild male Alpine ibex (*Capra ibex*), higher reproductive investment impairs ornament growth among adult cohorts [48], highlighting potential costs of current investment in reproduction on future reproduction success mediated by sexual ornament growth. However, this may not apply to between-cohort comparisons [150]. In female bighorn sheep (*Ovis canadensis*), horn size influenced reproductive success but not fecundity [50]. Important mediating factors, such as age [45], diet [151] and reproductive status [48] would probably have improved our analysis, but such information is not available in our population.

While our methods allowed live scoring of coat colours in the field without animal restraint, there are also some limitations. By customizing the colour scale for HK cattle and local field conditions, we limit our ability to understand the intermediate variations in coat colour pigmentation that may exist within the species. While similar scoring using five-colour charts has been conducted on artiodactyls [19], those assessments focused on comparing different species rather than exploring variability within a single species. Including HK cattle in species comparisons could also enhance our understanding of the mechanisms and adaptive significance of seasonal coat colour changes in subtropical ungulates.

Here, we present the first evidence of seasonal changes in coat colour of cattle, although the mechanisms behind this require further investigation. While we report coat colour changes in feral cattle, it is not possible to determine whether these seasonal changes also occurred in the population prior to them becoming feral between the 1950s and 1970s (figure 1). Additionally, the lack of studies in draught cattle [152], which are uncommon today, and limited knowledge of ‘yellow’ cattle phenotypes [58,61,153] further complicates our understanding of the basis of seasonal changes in coat colour of subtropical cattle. Identifying the genetic basis of coat colour plasticity in HK feral cattle (similar to [60]) could deepen our understanding of the mechanisms underlying colour adaptations in subtropical ungulates.

Our methods enabled us to score phenotypes non-invasively, with animals being neither restrained nor trapped to obtain any measurements. While this is essential to maintain good welfare in free-ranging populations [154,155], it may also cause uncertainty to some measurements. Notably, by scoring horn length as a categorical variable using visual comparison with ear length, we are unable to provide direct comparisons with other bovid species. However, as ear length in *Bos indicus* is known to be symmetrical (i.e. similar length of left and right ears [101]), varies by breed but not by age or sex [147] and is only weakly correlated to body size [156], we can assume that within-breed comparisons are valid for cattle. Hence, our analysis of inter-individual differences in horn length based on ear length comparison in HK cattle are likely to be valid, although they cannot be used for direct comparisons with other cattle populations.

## 5. Conclusion

The HK feral cattle are free roaming in a subtropical environment. We provide the first assessment of the seasonal changes in coat colour and sexual dimorphism in horn and body size of the HK feral cattle. Extreme coat colours are less common and susceptible to seasonal changes, with paler individuals in the wet season and darker ones in the dry season, suggesting plastic phenotypes. The main driver of seasonal coat colour appears to be thermoregulation. As cattle are sensitive to heat stress, understanding the mechanism through which mismatched phenotypes remain (e.g. darker individuals with higher thermoregulation costs in the wet season) has consequences for management of ruminants in changing environments. Additionally, with the global shift in climate, understanding the drivers of phenotypic plasticity in subtropical cattle improves our knowledge of adaptation in tropical and subtropical free-ranging populations in increasingly warm and unpredictable habitats.

## Data Availability

The datasets supporting this article have been uploaded to the Open Science Framework [100]. Supplementary material is available online [157].
